# *Streptococcus pneumoniae* Induces Autophagy through the Inhibition of the PI3K-I/Akt/mTOR Pathway and ROS Hypergeneration in A549 Cells

**DOI:** 10.1371/journal.pone.0122753

**Published:** 2015-03-24

**Authors:** Pu Li, Jing Shi, Qiao He, Qin Hu, Yun Ying Wang, Li Jun Zhang, Wai Ting Chan, Wei-Xian Chen

**Affiliations:** 1 Department of Laboratory medicine, the Second Hospital Affiliated to Chongqing Medical University, Chongqing, China; 2 Department of Laboratory medicine, the First Hospital Affiliated to Chongqing Medical University, Chongqing, China; 3 Centro de Investigaciones Biológicas, Consejo Superior de Investigaciones Científicas, Ramiro de Maeztu 9, Madrid, Spain; University of Kentucky, UNITED STATES

## Abstract

The present study focused on the action mechanism of *S*. *pneumoniae* (Sp) in inducing autophagy in human alveolar epithelial cells. Sp, a gram-positive extracellular bacterium, activates autophagy with considerably increased microtuble-associated protein light chain 3 (LC3) punctation in A549 cells. The accumulation of typical autophagosomes and conjugation of LC3 to phosphatidylethanolamine were observed in Sp-infected cells as an indication of autophagy. Using the pneumolysin (PLY) mutant, we successfully demonstrated that PLY is involved in initiating autophagy without affecting the expression levels of PI3K-III and Beclin1. PLY-mediated autophagy depends on the inhibition of the phosphoinositide 3-kinase/Akt/mammalian target of rapamycin (PI3K/Akt/mTOR) pathway. Furthermore, Sp could also lead to the reactive oxygen species (ROS) hypergeneration in A549 cells. Taken together, Sp infection-induced autophagy is PLY-mediated through ROS hypergeneration and mTOR inhibition. PI3K-I and rapamycin (autophagy inducers) enhanced bacterial clearance, whereas wortmannin (autophagy inhibitor) and acetylcysteine (ROS inhibitor) reduced intracellular bacteria clearance. Thus, Sp-induced autophagy represents a host-protective mechanism, providing new insight into the pathogenesis of respiratory tract Sp infection.

## Introduction

Extracellular bacterium *S*. *pneumoniae* (Sp) is a major human respiratory tract pathogen with a redundant set of virulence factors against host clearance [1]. One of the most important toxins released by Sp is pneumolysin (PLY), which has various immunomodulatory effects, including induction of cytokine production, reactive oxygen species (ROS) accumulation, and activation the classical pathway of complement [2–3]. Recent studies have shown that epithelial cells of the human respiratory tract and lung play a critical role in defending against host mucosal pathogens [4], but their role in fighting against Sp remains to be fully defined.

Autophagy is an intracellular process that delivers cytoplasmic components to the autophagosome and lysosome for degradation [5]. The autophagosome is the central organelle that eliminates intracellular pathogens and degrades cytoplasmic material to fuel starving cells [6]. The growing body of research has demonstrated that the autophagy pathway is a critical cellular process that strongly influences the functions of epithelial and immune cells [7]. Several signaling pathways have been implicated in regulating autophagy, including phosphoinositide 3-kinase/Akt/mammalian target of rapamycin (PI3K/Akt/mTOR) and ROS. Class I PI3Ks (PI3K-I) inhibits autophagy through triggers the target of mTOR (rapamycin) [8], whereas ROS upregulates autophagy under oxidative stress and inflammatory conditions, such as pathogenic microbe infections [9–10]. Thus, targeting critical autophagy regulators with a goal to promote autophagy in epithelial cells is an attractive new therapeutic strategy for mucosal pathogen infections [11–12].

Previous studies showed that the induction of autophagy can protect alveolar epithelial cells from respiratory pathogens infection, such as *Mycobacterium tuberculosis*, group A streptococci, and *Pseudomonas aeruginosa* [13–15], indicating that autophagy acts as an immune effector that mediates pathogen clearance [16]. However, most studies of bacterial autophagy only involve intracellular pathogens [17]. Until now, the role of autophagy in Sp pathogenesis has been completely unknown. Thus, we studied autophagy in Sp-infected A549 cells and, for the first time, revealed the induction of autophagy by pneumococcal PLY through inhibition of the PI3K/AKT/mTOR pathway via ROS. This observation could provide useful information for further understanding of the role of autophagy in respiratory pneumococcal infection and improve our knowledge of mucosal immunity against this pathogen.

## Materials and Methods

### Cells, bacteria, vectors, and cell transfection

A549 (human alveolar epithelial) cell lines and breast cancer cell line MCF7 were purchased from ATCC (USA) and maintained according to the supplier’s instructions. Bacteria strains Sp strain 35A (st35A) wild-type (WT) was isolated and collected from the Department of Laboratory Medicine (The Second Hospital Affiliated to Chongqing Medical University, Chongqing, China). Corresponding PLY-negative mutants (mut-PLY) developed through insertion-duplication mutagenesis, as described previously [18], were cultivated prior to infection analyses under antibiotic pressure with 10 mg/L erythromycin and 50 mg/L kanamycin. The plasmid pMV158GFP, which harbors the gene encoding the green fluorescent protein under the control of a promoter inducible by maltose, was a gift from Manuel Espinosa (Centro de Investigaciones Biológicas, Consejo Superior de Investigaciones Científicas, Spain) [19]. The pMV158GFP was transferred into Sp (Sp-GFP) according to the standard transfer assays as previously described [20]. The GFP-LC3 plasmid was kindly provided by Dr. Juan Chen (Chinese University of Hong Kong, China). The RFP-PLY plasmid was constructed by cloning the coding sequence of PLY (GenBank: X52474.1) to vector pHcRed1-N1/1 (Clontech, Cat. No. 632424), and the PCR primers for PLY were ATGGCAAATAAAGCAGTAAA (forward) and CTAGTCATTTTCTACCTTAT (reverse). A549 cells were transfected/co-transfected with GFP-LC3 and/or RFP-PLY plasmids using Lipofectamine 2000 reagent (Invitrogen, Grand Island, NY, USA) in serum-free Dulbecco's modified Eagle's medium (DMEM) (Invitrogen) following the manufacturer’s instructions.

### Proteins, antibodies, and reagents

Anti-PLY antibody was purchased from Abcam (ab71810, Cambridge, MA, USA), and sequestosome (p62) monoclonal antibody (H00008878-M01) was purchased from Abnova (Taipei, Taiwan). Phospho-PI3 kinase p85 (Tyr458)/p55 (Tyr199) antibody (4228), PI3 kinase class III antibody (3811), phospho-Akt (Ser473) (D9E) XP rabbit mAb (4060), phospho-ULK1 (Ser317) (D2B6Y) rabbit mAb (12753), mTOR rabbit mAb (2983), phospho-mTOR antibody (2974), phospho-Beclin-1 (Ser93/96) antibody (12476), and beclin-1 (D40C5) rabbit mAb (3495), rabbit monoclonal antibody against β-actin were all purchased from Cell Signaling Technology (Beverly, MA, USA). Rabbit polyclonal antibody against LC3B (light chain 3B, LC3B; L8918), rapamycin (R8781; Rapa, mTOR inhibitor), 3-methyladenine (M9281; 3-MA, PI3K-III inhibitor), and wortmannin (W1628; WT) were all obtained from Sigma Life Science (Shanghai, China). PIK-93 (S1489; Class I PI3 Kinase inhibitor) was purchased from Selleck Chemicals (Houston, TX, USA).

### Infection experiments

The bacteria were grown for 15 hours in Todd-Hewitt medium supplemented with 1% yeast extract (THY medium; Oxoid, England) at 37°C and 5% CO_2_ with shaking. The bacteria were then pelleted by centrifugation at 5000 rpm and resuspended in 10 mL of fresh THY broth, in which they were allowed to grow until the mid-logarithmic phase [21]. Thereafter, the optical density (OD) at 600 nm was measured, and the OD was adjusted to 0.25 (0.1OD = 1×10^8^ cells/mL). Accuracy of the bacterial colony-forming units (CFUs) was verified by plate counts of serial dilutions.

The bacteria were washed with phosphate buffer saline (PBS) before being changed to antibiotic-free medium for infection. The bacteria then were added to cell cultures at a 1:30 multiplicity of infection (MOI). After 1 hour, the infected cells were washed 3 times with PBS, and antibiotics (100 μg/mL gentamicin and 250 mg/mL

Ceftriaxone Sodium) were added to kill extracellular bacteria. The cells were further cultured for the indicated time (post infection; specific time was described in results section). The extracellular bacterial clearance and invading bacteria inside the cells were determined using the CFU assay [13].

### Transmission electron microscopy analysis

Transmission electron microscopy (TEM) was employed to identify autophagosomes with modified Karnovsky’s fixative [22]. A549 cells were infected with bacteria at an MOI of 1:30 for 1.5 h. The cells were then fixed with 2.5% glutaraldehyde. Ultrathin sections were stained with uranyl acetate and lead citrate and the sections were then analyzed by TEM. All sections were observed with an H7600 electron microscope (Hitachi, Japan).

### ROS assay

Relative changes in intracellular ROS in A549 cells were monitored using a fluorescent probe, 20, 70-dichlorofluorescein diacetate (DCFH-DA) [23]. The cells were seeded and cultured on 96-well plates at a density of 1×10^4^ cells/well. The cells were incubated with 10 μmol/L of DCFH-DA for 30 min, washed 3 times with serum-free DMEM medium, and then infected with bacteria for 1 hour. Then the cells were subjected to fluorescence detection at the indicated times (30 min, 1 h, 2 h, and 3 h) in a FLX800TBI fluorescence analyzer (BioTek, USA) with excitation/emission set at 488/525 nm. The values were expressed as percentage of relative fluorescence compared to the control.

### Western blotting

The cells were lysed and protease inhibitor cocktail added to obtain the whole protein to be quantified. The lysates with protein loading buffer were boiled for 5 min. The supernatants were collected and 40 μg of each sample were loaded onto 15% SDS-PAGE gels and electrophoresed for protein resolution. The separated proteins were then transferred to polyvinylidene fluoride membranes (0.45 μm pore size; Millipore, Billerica, MA) and blocked for 2 h at room temperature. The PVDF membranes were incubated overnight at 4°C with primary antibodies diluted at 1:1,000 in PBS with 5% bovine serum albumin buffer. After washing 3 times with tris-buffered saline (TBS) with 0.5% (v/v) Tween, the membranes were incubated for 2 h at room temperature with horseradish peroxidase-conjugated secondary antibody (1:2,000 dilution). The immunoreactive bands were visualized by enhanced chemiluminescence reagents (Millipore).

### Statistical analysis

All experiments were performed in at least three independent assays. Data are presented as percentage change compared with the controls ± SD from the three independent experiments. Group means were compared by one-way analysis of variance (ANOVA) with SPSS software. Statistical significances were analyzed by the two-tailed unpaired Student’s *t*-test; *P*-values of less than 0.05 were considered statistically significant.

## Results

### Sp infection induces LC3 punctation in A549 cells

**Fig 1:**
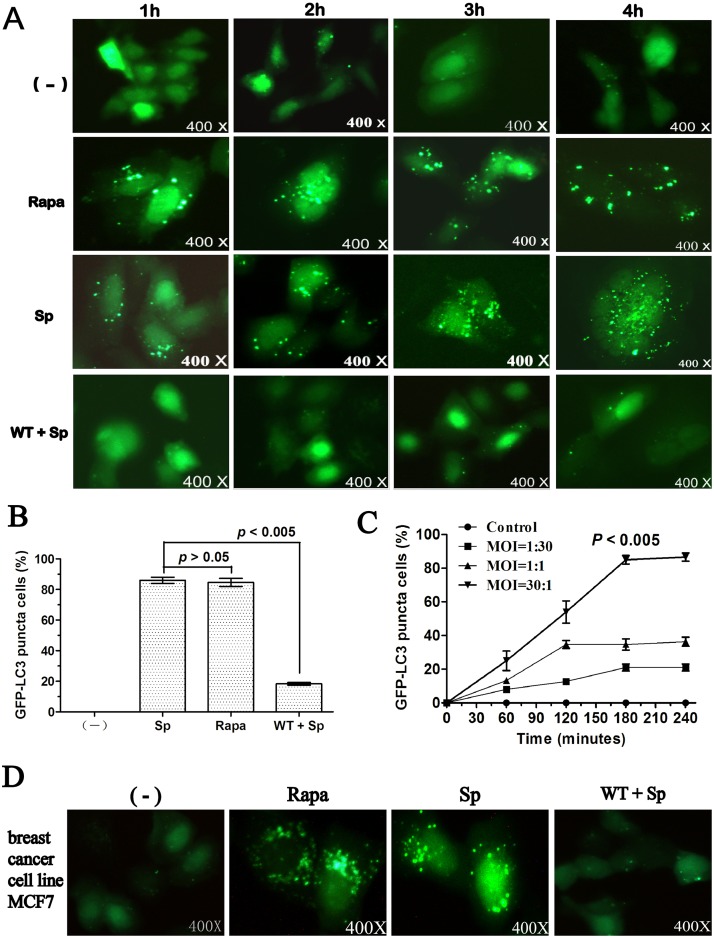
Sp infection induced LC3 punctation in A549 cells. A549 cells were infected with Sp (st35A) for 1 h (MOI=30:1). Before infection, the cells were treated with rapamycin (1 mM, 10 h) and wortmannin (2 μM, 2 h). (A) A549 cells were transfected with GFP-LC3 plasmids for 24 h. Then, the cells were infected with Sp for 1 h (MOI=30:1). (B) The puncta in each cell were counted and cells with more than 10 punctae were considered GFP-LC3 puncta cells. Values are from 100 cells/sample (one-way ANOVA; Tukey’s posthoc test). (C) A549 cells were infected at different times and with different MOIs. (D) Breast cancer cell line MCF7 cells were infected with Sp (st35A) for 4 h (MOI=30:1). The percentage of GFP-LC3 puncta cells was determined (one-way ANOVA; Tukey’s posthoc test). Data are representative of three experiments with similar results. Sp: *S*. *pneumoniae*; Rapa: rapamycin; WT: wortmannin.

To investigate whether infection by Sp (st35A) could induce autophagy, GFP-LC3 vectors were transfected into A549 cells. The A549 cells were infected with st35A, a strain of Sp, in a dose- and time-dependent manner. We noticed that Sp infection with an MOI of 30:1 (bacteria: cell) induced remarkably high LC3 punctation in A549 cells (Fig 1A). Cellular apoptosis was also detected to exclude the possibility that autophagy was caused by the induction of A549 cell death. LC3 punctation reached its peak at 3 h after infection and no observable cell death was detected at this time (Fig 1B). The LC3 punctation, thereafter, gradually decreased, probably because of increased autophagic cell death caused by infection [5] (Fig 1C). A549 cells transfected with GFP-LC3 were also treated with the autophagy activator rapamycin (positive) and inhibitor wortmannin (negative) as controls (Fig 1A). To determine whether autophagy is a general phenomenon in Sp infection, other cell type was also investigated. We also noted that Sp infection induced LC3 punctation in breast cancer cell line MCF7 (Fig 1D).

### Sp infection induces increased autophagosome and LC3-II formation and upregulated autophagic degradation

**Fig 2:**
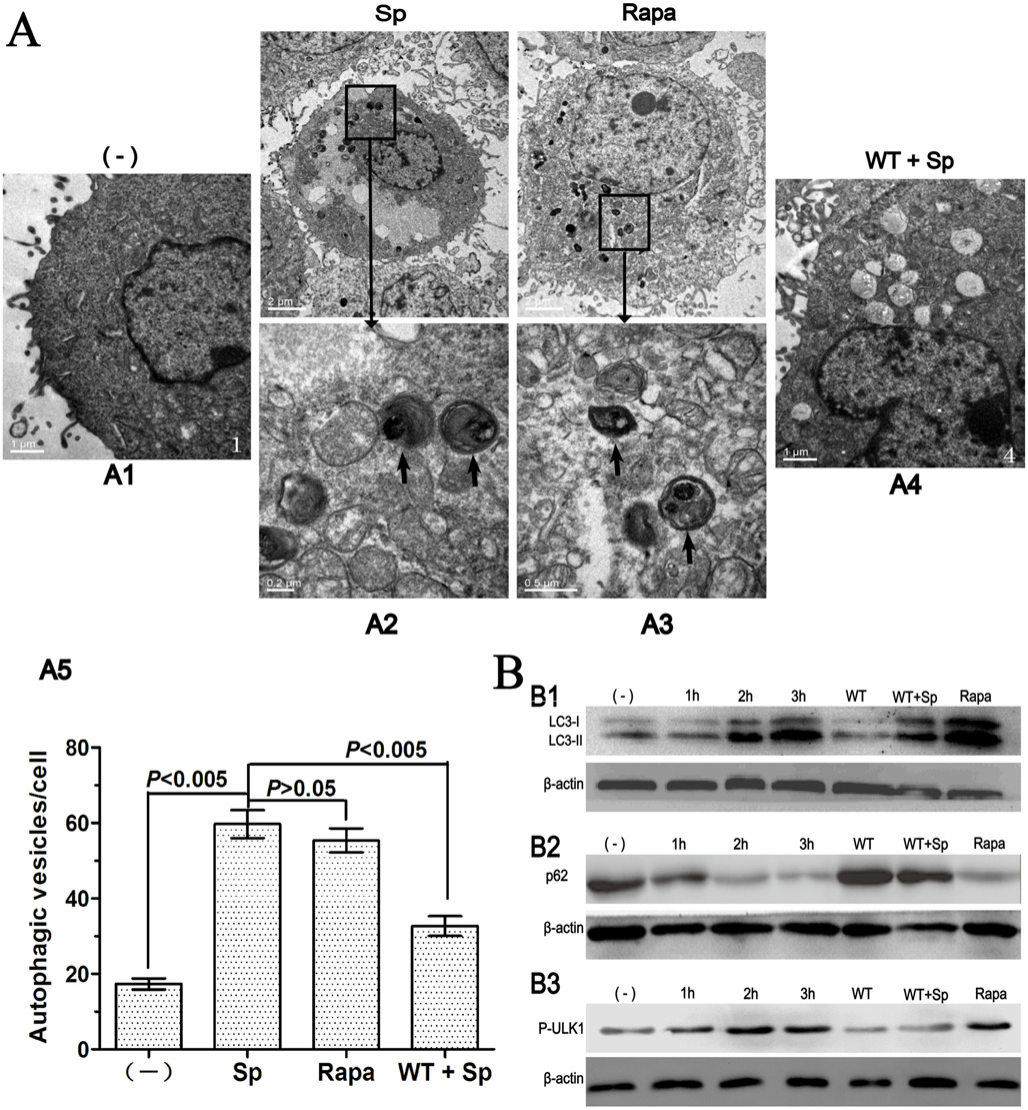
Sp infection induces increased autophagosome and LC3-II formation and upregulated autophagic degradation. (A) After 3 h infection, cells were processed and examined by TEM. A1. Untreated cells (–). A2. Cells infected with Sp (st35A). A3. Cells treated with rapamycin. A4. Cells treated with wortmannin and then infected with Sp. A5. The number of autophagic vesicles in each cell was determined with 10 cells in each sample (one-way ANOVA; Tukey’s posthoc test). (B) Sp infection induces LC3-II formation and upregulated autophagic degradation. B1. Western blot of LC3. B2. Western blot of P62. B3. Western blot of P-ULK1. Data are representative of three experiments with similar results. Sp: *S*. *pneumoniae*; Rapa: rapamycin; WT: wortmannin; p62: Sequestosome 1; p-ULK1: phosphorylated UNC-51-like kinase 1.

To exclude the possibility that the punctation flux was caused by transient overexpression of GFP-LC3 proteins, we further determined the newly formed autophagosomes with TEM. We observed that typical autophagosomes (with double membranes and vacuoles) were notably increased in st35A-infected A549 cells as compared with the control cells (Fig 2A). Thus, the morphological evidence further confirms that elevated autophagosome formation is consistent with the accumulation of LC3 punctation. Our results demonstrate that Sp infection-induced autophagy in A549 cells.

Autophagosomes accumulation might be caused by the blockage of autophagosome degradation rather than induction of autophagosome formation [24]. LC3-II is a well-established autophagosomes marker in mammalian cells. Therefore, we examined the change in LC3 patterns at the molecular level. Endogenous LC3-I transformation into phosphatidylethanolamine-conjugated (PE-conjugated) LC3-II was dramatically increased by Sp infection in a time-dependent manner (Fig 2B1). We monitored autophagic flux (refers to the whole process of autophagy, including autophagosome formation, maturation, fusion with lysosomes, subsequent breakdown and the release of macromolecules back into the cytosol) through the levels of endogenous p62 expression. Consistent with the induction of LC3-II accumulation, p62 showed a decrease by Sp infection in A549 cells (Fig 2B2). We also monitored phosphorylated UNC-51-like kinase 1 (p-ULK1). ULK1 can act as a convergence point for multiple signals that control autophagy [25]. Consistent with the induction of LC3-II accumulation, p-ULK1 showed an increase by Sp infection in A549 cells (Fig 2B3). Taken together, these findings firmly establish that Sp infection can specifically induce autophagy in A549 cells.

### Pneumococcal PLY is involved in initiating autophagy

Extracellular bacterium Sp could be phagocytosed by phagocytic cells [13–21], indicating autophagy through intracellular pathways. However, a previous study reported that even without bacteria internalization, cells were found to show significant autophagy and autophagy induction did not require intracellular bacteria [14]. Thus, it could be inferred that particular virulence factors released by Sp, such as pneumococcal PLY, attributed to autophagy induction.

**Fig 3:**
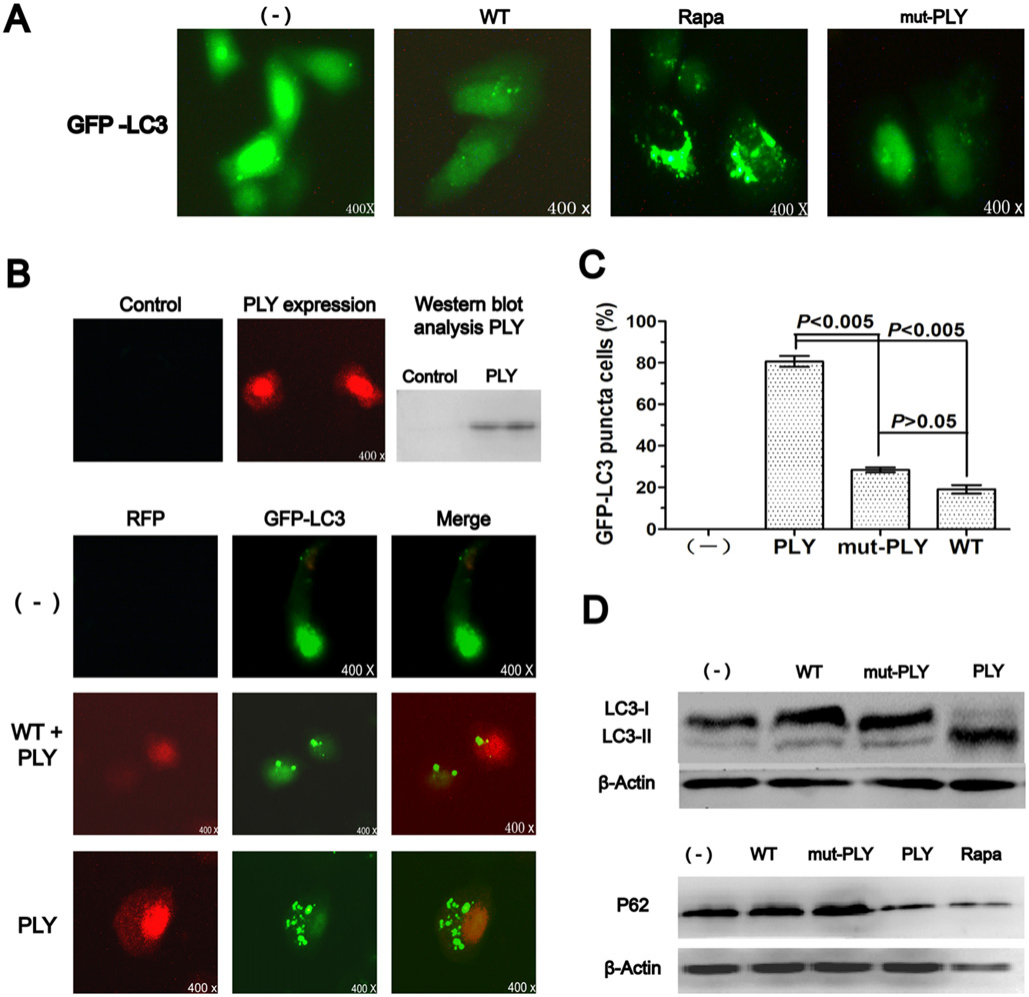
Pneumococcal PLY is involved in initiating autophagy. A549 cells were infected with Sp or mut-PLY for 1 h (MOI＝30:1). Before infection, the cells were also treated with rapamycin (1 mM, 10 h) and wortmannin (2 μM, 2 h). (A) mut-PLY failed to induce LC3 punctation. (B) RFP-PLY induced LC3 punctation in cotransfected cells. (C) The puncta in each cell were counted and cells with more than 10 punctae were considered GFP-LC3 puncta cells. Values are from 100 cells/sample (one-way ANOVA; Tukey’s posthoc test). (D) Western blotting of LC3 and p62. Data are representative of three experiments with similar results; Rapa: rapamycin; WT: wortmannin; p62: Sequestosome 1; PLY: pneumolysin; mut-PLY: PLY-deficient strain.

To determine PLY in inducing autophagy, we used the PLY-deficient strains (mut-PLY) and found that mut-PLY failed to induce LC3 punctation (Fig 3A). To further confirm whether intracellular PLY is involved in initiating autophagy, we constructed a RFP-PLY eukaryotic plasmid and transfected/cotransfected GFP-LC3 or/and RFP-PLY into A549 cells (Fig 3B). Autophagy typical markers for LC3 punctation, LC3 proteins, and p62 proteins were then examined as described above. We noticed that both the accumulation of LC3 punctation (Fig 3B and 3C) and the change in LC3 protein patterns (Fig 3D) were consistent with st35A-infected A549 cells. Thus, our results demonstrated that virulence factor PLY is involved in initiating autophagy in A549 cells.

### Pneumococcus PLY induces autophagy by inhibiting the PI3K/AKT/mTOR pathway and it is mediated through ROS hypergeneration

**Fig 4:**
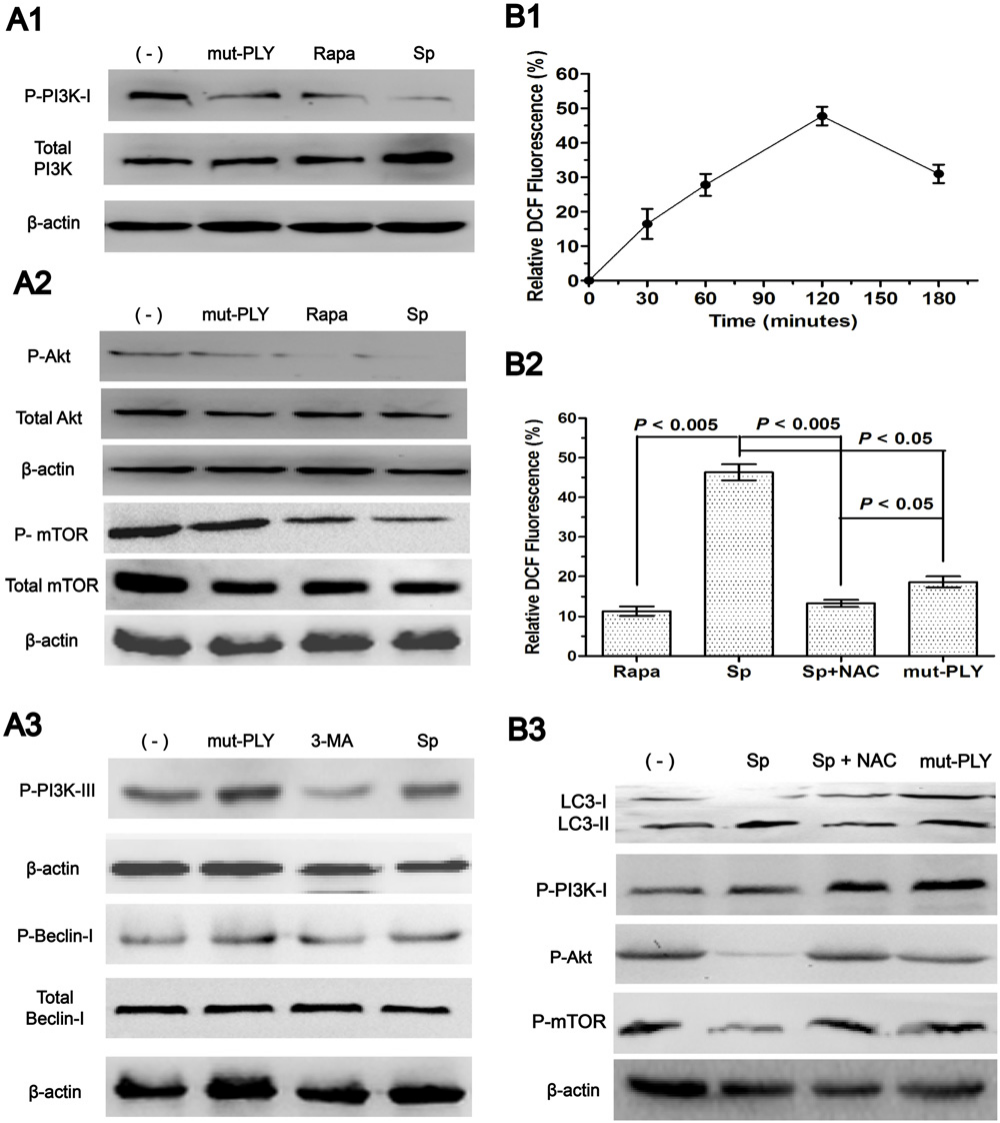
Pneumococcus PLY induces autophagy by inhibiting the PI3K/AKT/mTOR pathway and it is mediated through ROS hypergeneration. (A). Sp infection induced autophagy through the PI3K/Akt/mTOR inhibition pathway. A549 cells were infected with Sp for 1 h. A1. Western blotting of phosphorylated PI3K-I. Sp (st35A)-induced autophagy depends on P-PI3K-I inhibition. A2. Western blotting of phosphorylated Akt and mTOR. Sp inhibits Akt/mTOR signaling in A549 cells. A3. The expression levels of PI3K-III and Beclin 1 were not affected by Sp infection. (B). Sp infection induces ROS hypergeneration. A549 cells were infected with Sp for 1 h and intracellular ROS hypergeneration was observed in A549 cells. B1. A time-dependent intracellular ROS hypergeneration was observed in A549 cells infected with st35A. B2. ROS was significantly increased when the cells were infected with st35A (*p*<0.005). B3. The ROS-specific inhibitor NAC can upregulate PI3K/AKT/mTOR expression. Data are representative of three experiments with similar results. p-PI3K-I: phosphorylated PI3K-I; Rapa: rapamycin; Sp: *S*. *pneumoniae*; mut-PLY: PLY-deficient strain; ROS: reactive oxygen species; NAC: acetylcysteine (ROS inhibitor).

The PI3K pathways act as a regulator of autophagy [8]. Class I PI3Ks (PI3K-I) triggers the target of the rapamycin signaling pathway (PI3K/AKT/mTOR), which inhibits autophagy. Class III PI3Ks (PI3K-III) mediates the recruitment of specific autophagic effectors to the sites of origin of autophagic membranes and accelerates autophagy. Thus, we examined the involvement of autophagic protein PI3Ks. We found that PI3Ks, upstream regulators of autophagy, are involved in autophagy. Expression of phosphorylated PI3K-I (p-PI3K-I) was downregulated with Sp infection in A549 cells (Fig 4A1).

To dissect this pathway further, we examined a major downstream autophagy-related protein Akt and mTOR. As with p-PI3K-I, Sp infection also resulted in a substantial downregulation of phosphorylated Akt (p-Akt) (Fig 4A2). However, Sp infection induces the downregulation of the PI3K/AKT/mTOR pathway without affecting the expression levels of PI3K-III and Beclin 1 (Fig 4A3). From the present results, our data identified that Sp-induced autophagy depends on the PI3K/Akt/mTOR inhibition pathway in A549 cells.

To identify whether pneumococcus PLY-induced autophagy is mediated through ROS hypergeneration, we detected the intracellular levels of ROS in A549 cells. ROS are generated during mitochondrial oxidative metabolism, as well as in cellular response to xenobiotics, cytokines, and bacterial invasion. Growing bodies of study show that autophagy activated by ROS as a cytoprotective process [26–27]. On the other side, autophagy is stimulated to clear ROS to protect cells from bacterial toxin-induced damage [28]. However, the role of ROS generated by the host in pneumococcal containment during infection is unclear.

PLY induces the production of intracellular ROS in human neutrophils [2] and Sp may modulate the oxidative burst [29]. Therefore, the role of ROS in Sp**-**induced autophagy was examined. Sp infection increased the intensity of DCF fluorescence in A549 cells, showing that intracellular ROS was increased within 30 min and peaked at 120 min (Fig 4B1). Thereafter, a decline in ROS generation was observed.

To analyze whether pneumococcus PLY-induced autophagy is mediated through ROS hypergeneration, the cells were also treated with mut-PLY and analyzed for cell ROS formation. The levels of ROS in mut-PLY-treated A549 cells were significantly reduced (Fig 4B2). However, acetylcysteine, the ROS specific inhibitor, can block this effect and upregulation of the PI3K/AKT/mTOR expression (Fig 4B3). These results confirm that the PLY-induced autophagy is mediated through ROS hypergeneration.

### Autophagy regulates Sp clearance in A549 cells

**Fig 5:**
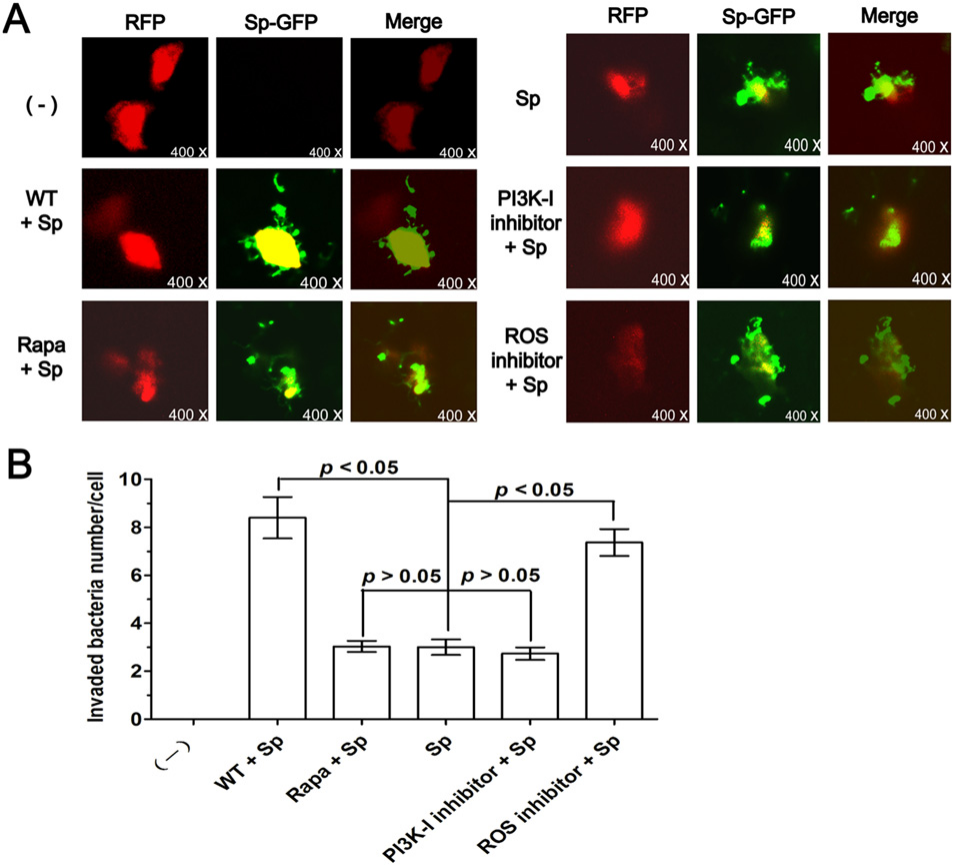
Autophagy regulates Sp clearance in A549 cells. (A) A549 cells were transfected with RFP-plasmid for 24 h and then infected with Sp-GFP for 1 h (MOI = 30:1). Before infection, the cells were also treated with rapamycin (1 mM, 10 h), wortmannin (2 μM, 2 h), PI3K-I inhibitor, or ROS inhibitor. The fluorescence images are displayed to confirm the internalization of bacteria. (B) Number of internalized bacteria per cell. CFU experiments were utilized to count the invading bacteria inside the cells. The data are representative of 100 cells (one-way ANOVA; Tukey’s posthoc test). Data are representative of three experiments with similar results. Rapa: rapamycin; WT: wortmannin; Sp: *S*. *pneumoniae*; ROS: reactive oxygen species; Sp-GFP: Sp + pMV158GFP plasmid; CFU: colony-forming unit.

Pneumococcus has long been considered an extracellular pathogen, and most autophagy studies thus far have only involved intracellular pathogens. To define the role of autophagy, we infected A549 cells with Sp-GFP to monitor internalization and bacterial clearance. After the cells were transfected with RFP-plasmid and then Sp infection, we utilized CFU to count the invading bacteria inside the cells. This approach showed bacterial internalization and the outline of cells (Fig 5), as well as the number of invading bacteria. To determine the role of autophagy in regulating phagocytosis of Sp and clearance, A549 cells were pre-treated with rapamycin, wortmannin, PI3K-I inhibitor, and ROS inhibitor respectively before infection. The intracellular Sp-GFP count decreased in rapamycin-treated and PI3K-I inhibitor-treated A549 cells, indicating that induction of autophagy can increase host defense against this pathogen. By contrast, the number of intracellular bacteria was increased by blocking autophagy with wortmannin in A549 cells. In particular, ROS-inhibitor treatment resulted in a marked increase in intracellular bacteria (Fig 5A and 5B). The data indicate that blocking autophagy with wortmannin or ROS inhibitor reduced Sp bacterial clearance. An autophagy activator, rapamycin, however, produced the opposite effect, improving bacterial clearance. Collectively, our observations indicate that autophagy might be a major benefit to host defenses by augmenting bacterial clearance.

## Discussion

In the present study, we addressed that Sp infection specifically induces autophagy through the PI3K/AKT/mTOR autophagy pathway and ROS hypergeneration in a human pulmonary epithelial cell line (A549). Moreover, our observations showed that the autophagy was initiatized by PLY, an important virulence factor of Sp. Bacterial pore-forming toxins have been shown to induce autophagy through mTOR [30]. Several pathogens, such as intracellular bacteria and viruses, could induce autophagy, although the pathways involved and the impact on infection outcomes vary with intracellular pathogens [24, 31–33].

Sp is traditionally considered an extracellular pathogen, and many virulence factors, such as PLY and capsular polysaccharide, might contribute to the extracellular pathogenic features of the bacterium [2, 34]. To our knowledge, prior to the current study, it was not known whether Sp-infection could induce autophagy. This is the first to demonstrate the induction of autophagy by Sp through the inhibition of the PI3K/Akt/mTOR pathway and ROS hypergeneration, as well as its physiological significance in relation to improved bacterial clearance.

Under stress or infection conditions, endogenous LC3-I proteins can conjugate with PE to form LC3-II [35]. Contrary to the cytoplasmic localization of LC3-I, LC3-II associates with both the outer and inner membranes of the autophagosome, thereby being a typical marker of autophagy formation. We identified LC3 punctation and confirmed the significant elevated autophagosome formation in st35A-infected A549 cells (Fig 1A; *p*<0.005). Since p62 accumulates when autophagy is inhibited, and decreased levels can be observed when autophagy is induced, p62 could be used as a marker to monitor autophagic flux. However, there is not always a clear correlation between increases in LC3-II and decreases in p62. Thus, we used LC3-II turnover in combination with p62 to monitor autophagy flux (Fig 2B). The increased autophagosome formation was also shown to be a result of autophagy induction rather than of blocked lysosome degradation. Rapamycin, a widely used inducer for autophagy induction, caused similar autophagy. However, wortmannin, a typical autophagy inhibitor, reduced autophagy following Sp infection.

Among the main mechanisms in the regulation of autophagy are the PI3Ks [8]. There are three distinct classes of PI3Ks; however, only Class I and III PI3Ks have been shown to play a role in autophagy [36]. The Class I and III PI3Ks have opposing roles in autophagy. PI3K-III mediates the recruitment of specific autophagic effectors to the origin sites of autophagic membranes and, thereby, plays a positively role in canonical autophagy. By contrast, PI3K-I trigger the AKT/mTOR signaling pathway to inhibit autophagy.

In this work, we demonstrated that the crucial upstream autophagy regulator PI3K-I was inhibited by Sp infection (Fig 4A). To evaluate its key downstream autophagy-related protein, we examined the phosphorylated Akt and mTOR. We noticed that protein levels of the phosphorylated Akt and mTOR were decreased significantly (Fig 4B; *p*<0.005), which were consistent with PI3K-I expression patterns. To determine additional virulence factors in inducting autophagy, we used the PLY-deficient strain (mut-PLY) and found that whereas the mut-PLY strain failed to induce autophagy, the Sp strain did induce it (Fig 3). Our observations indicate that virulence factor PLY might involve autophagy induction. Taken together, our studies indicate that Sp infection specifically induces autophagy and that this induction depends on the PI3K/AKT/mTOR autophagy pathway.

Having successfully demonstrated that Sp infection caused autophagy through the PI3K-I/AKT/mTOR pathway in A549 cells, we addressed the problem of how Sp induced the autophagic phenomenon. PLY has been characterized as a potent virulence factor released by Sp [37], which could induce cell death by pore formation and toxin-induced apoptosis, but although its cytolytic effects are well understood, less is known about the interactions between this potent toxin and the phenomenon of autophagy [3]. Therefore, the capacity of this pneumococcal factor to induce autophagy was analyzed. In this study, PLY-negative mutant (mut-PLY) was developed through insertion-duplication mutagenesis; The RFP-PLY plasmid was constructed and transfected to A549 cells, then PLY was overexpressed intracellularly. We did not observe significant apoptosis-changes in cell morphology.

In our analyses, transfection of A549 with the plasmid RFP-PLY resulted in a significant induction of autophagy (Fig 3; *p*<0.005) and ROS hypergeneration (Fig 4), while the corresponding PLY-negative mutants led to a blockade of autophagy induction. Tattoli *et al* found that infection of epithelial cells with *Shigella* and *Salmonella* triggers acute intracellular amino acid starvation due to host membrane damage. Pathogen-induced amino acid starvation caused downregulation of mTOR activity, resulting in the induction of autophagy [38]. This study could be the most probable explanation for autophagy is induced in a PLY-dependent manner.

ROS has been shown to activate autophagy to protect cells from nutrient starvation, cell death, and invading pathogens. Some studies show that ROS activate autophagy by regulating mTOR activity. In malignant gliomas, ROS disrupt mitochondrial membrane potential and induce autophagy through inhibiting Akt/mTOR signaling [39]. In glioma cells, ROS induce autophagy by inhibiting mTOR in a BNIP3-dependent manner (BNIP3: BCL2/adenovirus E1B 19 kDa protein-interacting protein 3) [40]. ROS can also inhibit PTEN (phosphatase and tensin homolog located on chromosome 10) [41], which has been shown to activate autophagy through the downregulation of the PI3K-I/Akt pathway. However, many unknowns still exist regarding the downstream signals of ROS in autophagy regulation. In this study, we detected the hypergeneration of ROS and downregulation of PI3K-I/Akt/mTOR during Sp infection. Taken together, we conclude that pneumococcal PLY-induced autophagy is mediated through ROS hypergeneration.

Recently, the role of autophagy in bacterial infections disease has garnered increasing interest [42–43], and it has been shown to play crucial roles in host defense, especially in immunological cells. Moreover, autophagy has a direct impact on immunity and inflammatory response within the whole organism. Intracellular bacteria, such as *Salmonella* and *Mycobacterium tuberculosis*, have all been shown to fuse with or be directly engulfed by autophagosomes, offering an alternative method for the bacteria destruction by autophagy [44–45]. Although most studies of bacterial-specific autophagy involve the capture of intracellular bacteria, autophagy can similarly eliminate extracellular bacteria *P*. *aeruginosa*, which have the capacity of autophagy induction in the alveolar macrophage cell line MH-S [14, 33].

Our studies have also defined that human pulmonary epithelial cell lines A549 can capture and destroy the invading Sp (Fig 5). PI3K is a classic pathway to influence the whole process of autophagy [8], whereas ROS can upregulate autophagy when host cells were infected with pathogenic microbe [9–10]. Thus, we targeted critical autophagy regulators with its inhibitors to observe the autophagy impact on Sp infections. Treatment with the autophagy inhibitor wortmannin or ROS inhibitor resulted in a marked increase in bacterial load whereas treatment with the autophagy inducer rapamycin or PI3K-I inhibitor enhanced Sp clearance (Fig 5B). Because airway epithelial cells and alveolar epithelial cells play a pivotal role in orchestrating both the innate immune response and the transition to adaptive immunity [46–47], autophagy in epithelial cells could impact the consequence of Sp infection. Previous reports have showed that Sp could be killed in a lysosome dependent manner [48], but host cells may use more than one strategy to eliminate invading pathogens. Based on our data, the mechanism of autophagy also plays a role in limiting Sp infection. In fact, lysosome system overlaps autophagy. More researches should be taken out in this field. Our results suggest that autophagy can mediate the removal of Sp in alveolar epithelial cells. Thus, Sp could serve as a model pathogen for investigating the impact of autophagy on crucial cellular events, such as bacterial entry and clearance. But Sp infection-induced autophagy in vivo need further assessment, however, this observation could provide useful information for further understanding the role of autophagy in respiratory pneumococcal infection and ultimately lead to new therapeutic targets.
